# Aerosolization behavior of antimicrobial resistance in animal farms: a field study from feces to fine particulate matter

**DOI:** 10.3389/fmicb.2023.1175265

**Published:** 2023-04-20

**Authors:** Huibo Xin, Tianlei Qiu, Yajie Guo, Haoze Gao, Liqiu Zhang, Min Gao

**Affiliations:** ^1^Beijing Key Laboratory of Agricultural Genetic Resources and Biotechnology, Institute of Biotechnology, Beijing Academy of Agriculture and Forestry Sciences, Beijing, China; ^2^Beijing Key Laboratory for Source Control Technology of Water Pollution, Engineering Research Center for Water Pollution Source Control and Eco-remediation, Beijing Forestry University, Beijing, China

**Keywords:** animal farms, fine particulate matter, antibiotic resistance, human pathogenic bacteria, aerosolization behavior

## Abstract

Antibiotic resistance genes (ARGs) and antibiotic-resistant bacteria (ARB) in animal feces can be released into the atmosphere *via* aerosolization, posing a high health risk to farm workers. So far, little attention has been paid to the characterization of the aerosolization process. In this study, fecal and fine particulate matter (PM2.5) samples were collected from 20 animal farms involving swine, cattle, layers, and broilers, and the ARGs, ARB, and human pathogenic bacteria (HPB) were loaded in these two media. The results showed that approximately 70% of ARGs, 60% of ARBs, and 43% of HPBs were found to be preferential aerosolization. The bioaerosolization index (BI) of target 30 ARGs varied from 0.04 to 460.07, and the highest value was detected from *tetW*. The highest BI values of erythromycin- and tetracycline-resistant bacteria were for *Kocuria* (13119) and *Staphylococcus* (24746), respectively, and the distribution of BI in the two types of dominant ARB was similar. Regarding the bioaerosolization behavior of HPB, *Clostridium saccharolyticum* WM1 was the most easily aerosolized pathogen in swine and broiler farms, and *Brucella abortus* strain CNM 20040339 had the highest value in cattle and layer farms. Notably, the highest BI values for ARGs, ARB, and HPB were universally detected on chicken farms. Most ARGs, ARB, and HPB positively correlated with animal age, stocking density, and breeding area. Temperature and relative humidity have significant effects on the aerosolization behavior of targets, and the effects of these two parameters on the same target are usually opposite. The results of this study provide a basis for a better understanding of the contribution of animal feces to airborne ARGs and HPBs in farms, as well as for controlling the transport of the fecal microbiome to the environment through the aerosolization pathway.

## 1. Introduction

The expansion of centralized animal feeding operations (CAFOs) is one of the main causes of increased environmental antimicrobial resistance (AMR) (Zhu et al., 2013). Globally, 73% of all antimicrobials sold on Earth are used in animals raised for food (Van Boeckel et al., 2019), and this value accounts for approximately 80% in some developed countries such as Canada and the US (Tang et al., 2017; Xiong et al., 2018). The total annual consumption of antibiotics in animal husbandry in China is 97,000 tons (Song et al., 2020), ranking first worldwide. The abuse of antibiotics promotes the development of AMR and the emergence of novel resistance mechanisms in farmed animals (Van Boeckel et al., 2019). As an important AMR reservoir (Xie et al., 2019), animal gut flora are continuously released into the environment through excreta (mainly feces), and the animal-related AMR elements contained therein would pollute terrestrial and aquatic ecosystems (Zhao et al., 2021), including soil (Yang et al., 2022), groundwater (Zainab et al., 2020), and crops (He et al., 2016).

In recent years, the air has been proven to be an important pathway for AMR transmission from livestock to humans (de Rooij et al., 2019) through exposure to fecal ARGs *via* dust (Munk et al., 2018). Recent research has indicated that the structure of the antibiotic resistome in animal feces is correlated with that in farm dust (Luiken et al., 2020), and the average contribution rate of pig manure to airborne bacteria varies seasonally (Song et al., 2021; Bai et al., 2022). To date, studies on the connection between AMR in feces and air have focused on their communities (resistomes and bacterial microbiomes), and few studies have explored this relationship based on individual targets. This limits our understanding of the aerosolization process of specific AMR elements in feces, which is of great significance for certain worrying and high-risk ARGs, ARBs, and HPBs.

The aerosolization behavior was used to evaluate the microbial release ability of solids or liquids (Moletta et al., 2010). The earliest study on bacterial aerosolization was conducted using the ratio of the concentration of culturable bacteria in the air to that in natural waters (Parker et al., 1983). Although preferential aerosolization has been detected at the bacterial phylum level (Moletta et al., 2007), the aerosolization behavior of bacterial species is influenced by multiple factors (Parker et al., 1983). Recently, to evaluate the effectiveness of dissemination, an increasing number of researchers have begun to focus on the aerosolization behavior of microorganisms in biological contamination sources, including solid waste compost (He et al., 2019), animal manure compost (Wang R. et al., 2022), and sludge compost (Lu et al., 2021). However, there have been no studies on bacterial escape from animal feces, particularly regarding the aerosolization behavior of AMR elements and their associated factors.

In this study, animal feces were assumed to be the main source of airborne AMR in CAFOs. A systematic study was conducted on the aerosolization behavior of ARGs, ARBs, and HPBs and the corresponding potential factors from feces to PM2.5. The aim was to describe the overall characteristics of the animal-specific aerosolization behavior of target elements, determine the preferential aerosolization of targets based on their bioaerosolization indices, and explore the key parameters that affect aerosolization behavior.

## 2. Materials and methods

### 2.1. Sample collection

In this study, fecal and PM2.5 samples were collected in the Pinggu, Miyun, and Huairou districts of Beijing, China. In total, 40 samples (20 fecal and 20 air) were collected from four types of animal farms (swine, cattle, layer, and broiler) and five farms for each animal species. The animal breeding houses were semi-enclosed with natural ventilation. Information on animal breeding and environmental indices was recorded while collecting the samples, and the details of the sampling site were described in a previously published article (Xin et al., 2022).

The fecal samples were collected using a 5-point sampling method, with 10 g of feces at each point, and a total of 50 g of feces were collected from each farm and mixed well. PM2.5 samples (1.5 m above the ground) were collected using medium-volume (100 L/min) air samplers (2030A, Laoying, Qingdao, China). The particle separation device [with a 50% cut point diameter (Da50) = 2.5 ± 0.2 μm] allowed particulate matter with aerodynamic diameters of ≤2.5 to be collected on the filters. The sampling time was approximately 48 h. The blank filters used for sampling were 90 mm micro-quartz fiber filters (Ahlstrom Munktell, Falun, Sweden), and the filters were dried and sterilized in a muffle furnace at 400 °C for 5 h before sampling. The collected feces and filters were placed in an ice box and returned to the laboratory within 5 h and stored at −80 °C. A particle mass counter (MetOne, OR, USA) was used to measure the PM2.5 mass concentration, temperature, and relative humidity of air in the breeding house.

For culturable airborne ARBs, an Anderson six-stage sampler was used to collect aerosols 1.5 m above the ground inside the breeding houses. The device contains 400 pores at each stage, with pore size decreasing from > 7.0 μM at stage I to 4.7–7.0 μM at stage II, 3.3–4.7 μM at stage III, 2.1–3.3 μM at stage IV, 1.1–2.1 μM at stage V, and 0.65–1.1 μM at stage VI. Aerosols collected during stages IV–VI were considered to be fine particulate matter (Gao et al., 2015). Samples for the analysis of antibiotic-resistant bacteria were collected for 2 min, at 28.3 L/min. The sampling pump was calibrated using a flowmeter (Yuyao, Zhejiang, China) before sampling. Air samples were collected on two types of media (20 ml) simultaneously, including LB nutrient agar enriched with tetracycline or erythromycin. Tetracycline was dissolved using ultrapure water and filtered through a 0.22 μM PES filter, and erythromycin was dissolved using 95% ethanol and filtered through a 0.22 μM Nylon filter. The corresponding antibiotic solutions were added to the sterilized LB nutrient agar medium, and the working concentrations of tetracycline and erythromycin were 16 and 48 mg/L, respectively (Yang et al., 2016). Three parallel samples were collected for each medium type. Dishes were then sealed, transported immediately to the laboratory, and cultured for 48 h at 28 °C.

### 2.2. DNA extraction and droplet digital PCR

For PM2.5 samples, one-eighth of a micro-quartz fiber filter was added into 50 ml of sterile 1 × PBS buffer and centrifuged at a low speed of 200 *g* at 4 °C for 3 h. After vortex shaking, the resuspension was filtered through a 0.2 μM PES filter membrane, and DNA was extracted from the particulate matter on this membrane (Jiang et al., 2015). For the culturable total and antibiotic-resistant bacteria, all colonies from three replicates were scraped down, transferred into 15 ml sterile centrifuge tubes containing PBS buffer, and centrifuged at 12,000 rpm for 1 min. DNA was extracted from 300 μl of the resulting pellet. For fecal samples, 0.5 g of well-mixed fecal samples were considered for DNA extraction from the corresponding animal feces. DNA extraction for the above samples was performed using FastDNA^®^ SPIN Kit for Soil (MP Biomedicals, CA, USA). The concentration and purity of the extracted DNA were determined by Qubit^®^ dsDNA High Sensitivity Assay Kit (Thermo Fisher Scientific, USA).

To characterize the profiles of ARGs in the fecal and air samples, the concentrations of 16S rRNA and 30 subtypes of ARGs encoding resistance to eight antimicrobial classes (Xin et al., 2022) were performed by the QX200 Droplet Digital™ PCR system. The annealing temperatures of ddPCR, sequences of the forward and reverse primers, and resistance mechanism of the 30 ARGs have been reported in previous studies (Gao et al., 2018; Ding et al., 2020). The ddPCR and data collection processes were based on previously published articles (Gao et al., 2018; Ding et al., 2020; Xin et al., 2022). The abundance of each ARG was normalized to “relative abundance” (copies per 16S rRNA gene) by dividing the copy number of each ARG by the number of 16S rRNA genes.

### 2.3. Illumina sequencing and data processing

Using Illumina sequencing, the structures of the unculturable and culturable bacterial communities in fecal and PM2.5 samples were determined. The V3–V4 hypervariable region of the bacterial 16S rRNA was amplified with the universal primers 338F (5′-ACTCCTACGGGAGGCAGA-3′) and 806R (5′-GGACTACHVGGGTWTCTAAT-3′) using an ABI GeneAmp^®^ 9700 PCR thermocycler (ABI, CA, United States), and the amplification reaction and conditions were conducted following previous studies (Chen et al., 2019; Xin et al., 2022). PCR products were purified and sequenced on an Illumina PE300 platform (Majorbio, Shanghai, China). Sequencing data were analyzed following previously described approaches (Gao et al., 2018; Xin et al., 2022). Raw sequencing data were uploaded to the NCBI Sequence Read Archive (SRA) database with registration numbers SRP079945 for culturable ARBs and SRP079949 for HPBs.

### 2.4. Identification of human pathogenic bacteria

Human pathogenic bacteria in fecal and air samples were determined based on a database of bacterial pathogens reported in an earlier study (Chen et al., 2016), in which the 16S rRNA gene sequences of bacterial pathogens were all available from NCBI (http://www.ncbi.nlm.nih.gov/). The 16S rRNA gene sequences of each sample were blasted against those of bacterial pathogens in the database, with an E-value of < 1 × 10^−10^. The results of BLAST hits were then filtered to identify the HPB with a sequence similarity threshold of > 99%.

### 2.5. Data analysis

The bioaerosolization index (BI) was used to quantify the aerosolization behavior of the microorganisms. The BI calculation is presented in Eq. (1), which is a concentration factor (Moletta et al., 2010).


$$\begin{array}{l} BI\;=\;\frac{RA_{PM2.5}}{RA_{feces}}\;\left(1\right) \end{array}$$

where RA_*PM*2.5_ is the relative abundance of ARGs, ARBs, and HPBs in PM2.5, and RA_*feces*_ is the relative abundance of ARGs, ARBs, and HPBs in the fecal samples.

Heatmaps were conducted using R software (“vegan” and “pheatmap” packages). Line plots merged with scatter plots were drawn using the OriginPro 2022 software. Partial least squares path modeling (PLS-PM) was used to detect the causal relationships between the influencing factors and aerosolization behavior. All data were standardized before establishing a priori models using Z-scores in SPSS26.0. PLS-PM was conducted with the “plspm” package in the R software. PLS-PM analysis generally used goodness of fit (GOF) to judge the overall model fit: the larger the GOF value, the higher the model fit; GOF = 0.10, the model fit is low; GOF = 0.25, the model fit is medium; and GOF = 0.36, the model fit is high (Henseler and Sarstedt, 2013). For the correlation bubble plot, the package “vegan” was used to calculate “Spearman” correlations between environmental factors and BI values, and the package “ggplot2” was used to make the bubble plot.

## 3. Results and discussions

### 3.1. An abundance of antibiotic resistance genes, antibiotic-resistant bacteria, and human pathogenic bacteria

**Figure 1:**
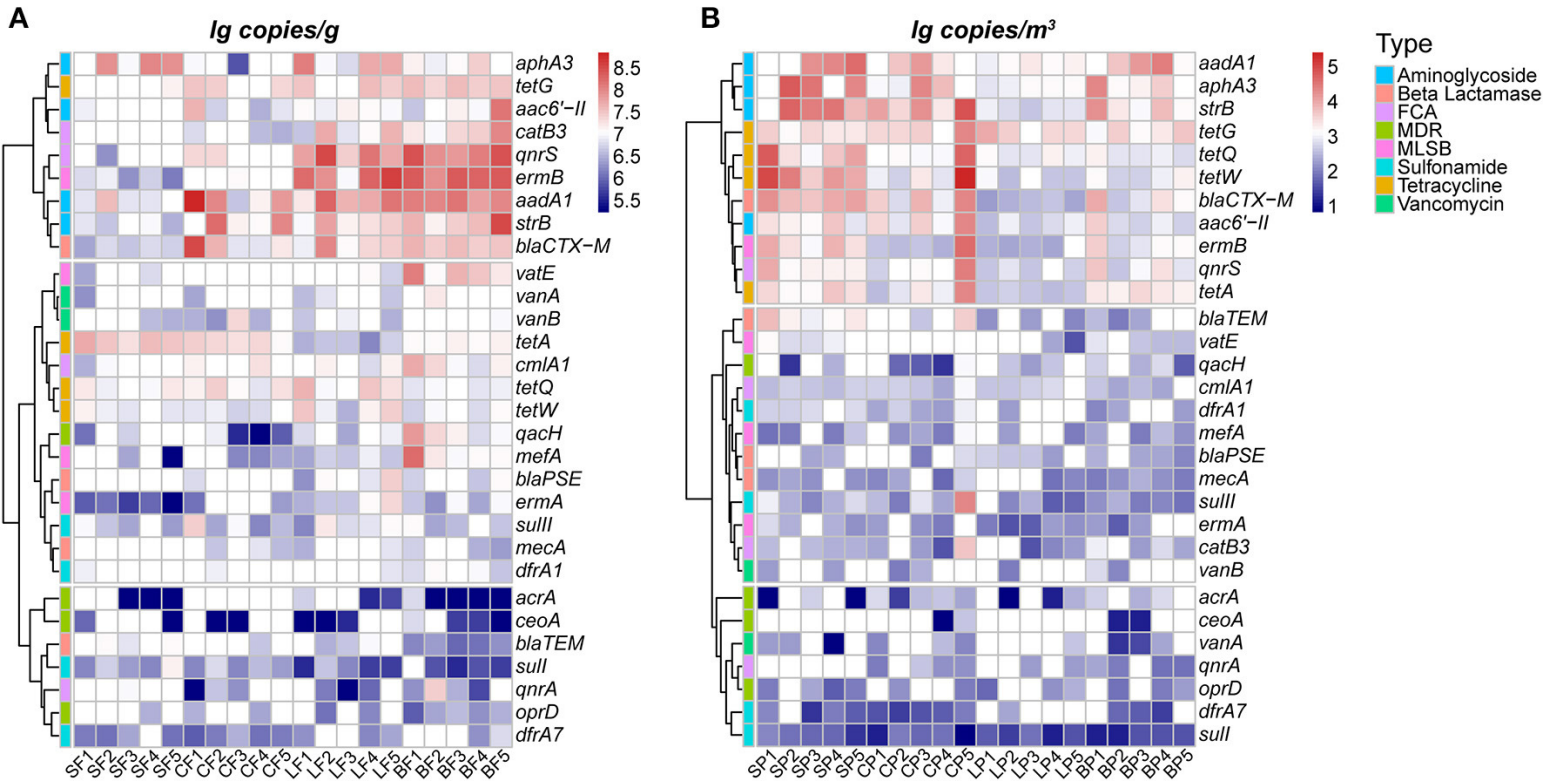
Abundance (log 10 transformed concentration) of 30 ARGs subtypes in animal fecal **(A)** and PM2.5 **(B)** samples by animal species. The first letter of the sample name represents the category of animals: S, swine; C, cattle; L, layer; B, broiler. The second letter represents the category of media: F, feces; P, PM2.5.

The bacterial biomass in animal feces ranged from 3.79 × 10^9^ to 2.40 × 10^10^ copies/g as indicated by the concentration of 16S rRNA, and the average concentration of 30 ARGs was 2.88 ± 1.92 × 10^7^ copies/g (Figure 1). Overall, ARGs encoding aminoglycoside resistance had the highest mean concentrations, with values of 6.46 ± 4.23 × 10^7^ copies/g, and the lowest mean concentration (4.84 ± 2.13 × 10^6^ copies/g) was observed for ARGs encoding resistance to multidrug-resistant classes. This distribution pattern is consistent with that of previous studies on swine feces (Zhang et al., 2019; Yue et al., 2021), poultry feces (McEachran et al., 2015), and cattle feces (Qian et al., 2018; Zhu et al., 2021; Yuan et al., 2022) because aminoglycosides are the most widely used antibiotics in the animal farming industry in China (Zhao et al., 2021). The concentrations of specific ARGs in the feces varied depending on the animal species; mean concentrations of ARGs in the feces of layer (2.66 ± 4.84 × 10^7^ copies/g) and broilers (3.65 ± 5.88 × 10^7^ copies/g) were significantly higher than those of swine and cattle (*p* < 0.05), which further confirmed the severity of AMR contamination in poultry farms (Cheng et al., 2013; Qian et al., 2018). In addition to the type and amount of antibiotics used in animal breeding processes, differences in the structure of the intestinal flora of animals may also play an important role in the different concentrations of fecal ARGs (Zhao et al., 2010).

In terms of PM2.5 samples, the mean concentrations of 16S rRNA gene and ARGs were 3.40 ± 2.04 × 10^5^ copies/m^3^ (ranged from 8.89 × 10^4^ to 1.84 × 10^6^ copies/m^3^) and 3.94 ± 1.73 × 10^3^ copies/m^3^ (from 8.33 × 10^0^ to 1.84 × 10^6^ copies/m^3^), respectively (Figure 1B). Similar distributions were also detected in pigs (Song et al., 2021), chicken (Xu et al., 2022), and cattle farms (Bai et al., 2022), with the highest and lowest concentrations of airborne ARGs detected from those encoded aminoglycosides (1.20 ± 0.61 × 10^4^ copies/m^3^) and multidrug resistance (2.5 ± 1.44 × 10^2^ copies/m^3^), respectively. The animal-specific concentration of airborne ARGs was observed in PM2.5 such that the highest mean value was also determined in layer (2.56 ± 1.13 × 10^4^ copies/m^3^) and broiler (5.64 ± 1.14 × 10^4^ copies/m^3^) farms. According to the results in Figure 1, these synchronous concentrations of ARGs in PM2.5 and fecal samples can be used to account for the contribution of feces to airborne ARGs in animal farms.

**Figure 2:**
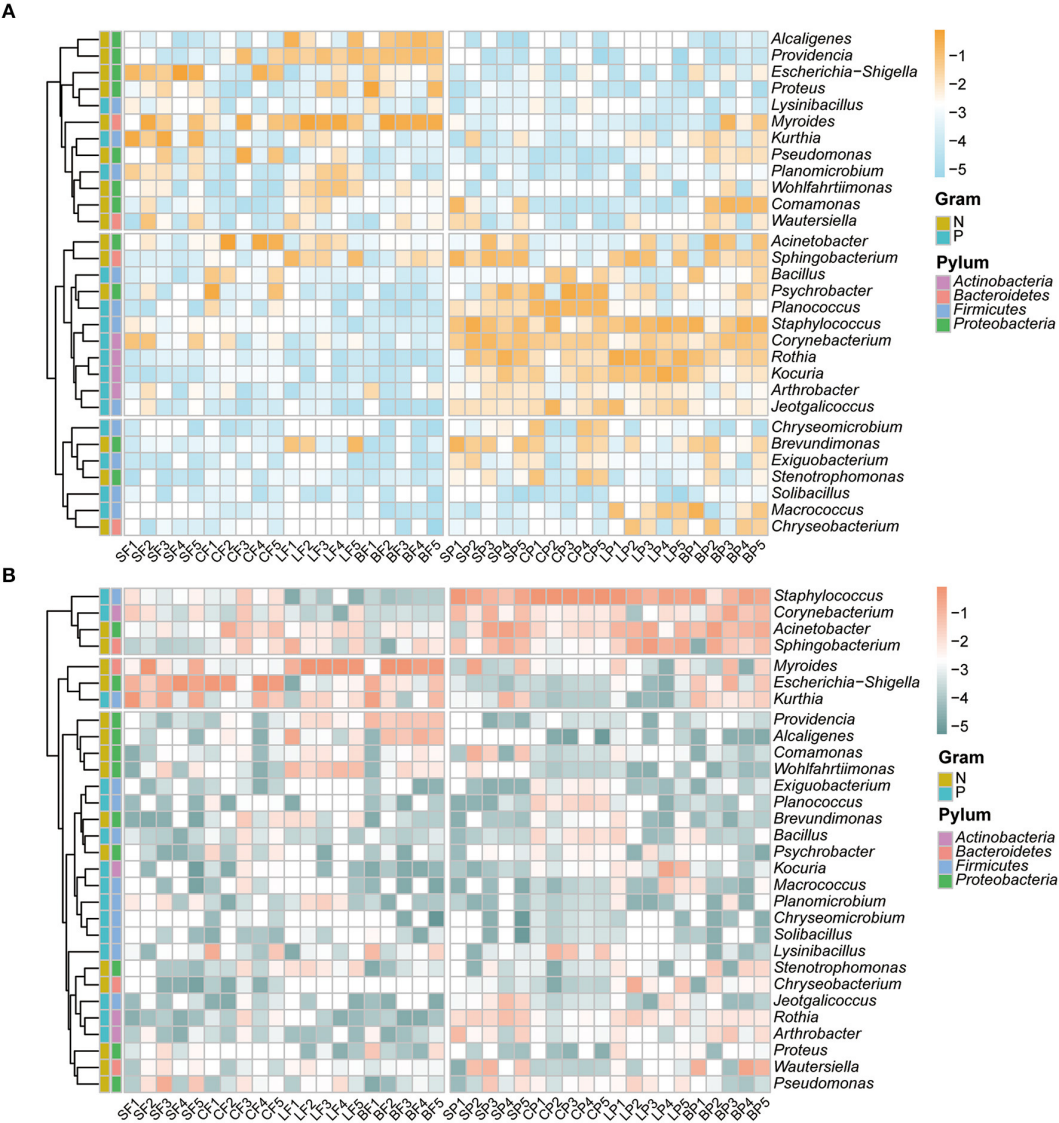
Relative abundance (log 10 transformed) of top 30 erythromycin- **(A)** and tetracycline-resistant bacterial genera **(B)** in feces and fine particles by animal species. The first letter of the sample name represents the category of animals: S, swine; C, cattle; L, layer; B, broiler. The second letter represents the category of media: F, feces; P, PM2.5.

The relative abundances of the top 30 erythromycin- and tetracycline-resistant bacterial genera in fecal and PM2.5 samples, respectively, were analyzed using a heatmap (Figures 2A, B). Overall, similar dominant genera with comparable relative abundances were detected in the two ARB types, either in the air or fecal samples. However, the distribution of dominant ARBs in the air differed from that in the fecal samples. The 30 most dominant erythromycin-resistant bacteria belonged to four bacterial phyla (Figure 2). For erythromycin-resistant bacteria, *Proteobacteria* (53.1 ± 13.0%) was the predominant bacterial phylum in fecal samples, followed by *Bacteroidetes* (28.3 ± 17.7%) and *Firmicutes* (15.6 ± 15.1%). The three dominant phyla for fecal tetracycline-resistant bacteria were also *Proteobacteria, Bacteroidetes*, and *Firmicutes* with similar relative abundances of 41.3 ± 19.3%, 37.5 ± 31.0%, and 17.0 ± 9.82%, respectively. *Firmicutes* (36.4 ± 12.8%) was the predominant erythromycin-resistant bacterial phylum in PM2.5 samples, followed by *Actinobacteria* (27.0 ± 18.2%), *Proteobacteria* (24.6 ± 14.5%), and *Bacteroidetes* (12.0 ± 8.1%). For tetracycline-resistant bacteria, *Firmicutes* (50.8 ± 28.6%), *Bacteroidetes* (19.6 ± 13.5%), *Proteobacteria* (18.5 ± 11.7%), and *Actinobacteria* (10.7 ± 6.2%) were the most dominant in the air. According to previous studies on the air environment of poultry and pig farms (Wang et al., 2019; Bai et al., 2022; Jezak and Kozajda, 2022), *Proteobacteria, Firmicutes*, and *Actinobacteria* were also detected as the dominant bacteria in feces or air using molecular biological methods. Although similar dominant phyla were detected between the air and fecal ARBs, there were differences in their specific relative abundances. This phenomenon has also been previously reported for *Firmicutes* and *Bacteroidetes* on layer and broiler farms (Hong et al., 2012; Wery, 2014).

As shown in Figure 2, *Myroides* and *Escherichia–Shigella* were the top two prevalent genera in fecal samples, and the third dominant genera among the erythromycin- and tetracycline-resistant bacteria was *Acinetobacter* (8.52 ± 2.09%) and *Kurthia* (10.4 ± 8.2%), respectively. However, distinctive dominant bacterial genera were found in PM2.5 samples that *Staphylococcus* was consistently detected as dominant ARBs in both erythromycin- and tetracycline-resistant bacteria with relative abundance of 15.6 ± 12.6% and 43.6 ± 28.5%, respectively. Previous studies on airborne ARBs have mostly focused on urban areas (Wang M. et al., 2022), which have also detected the prevalence of antibiotic-resistant *Staphylococcus* in the urban air of Beijing. As a bacterium that contains important potentially pathogenic species in humans and animals, the universal existence of antibiotic-resistant *Staphylococcus* in the air has given us a warning. The remaining prevalent genera for erythromycin-resistant bacteria were *Rothia* (11.5 ± 1.29%) and *Psychrobacter* (9.01 ± 1.47%). The tetracycline-resistant bacteria were *Acinetobacter* (13.4 ± 12.5%) and *Sphingobacterium* (14.8 ± 10.8%). Those genera, including *Bacillus, Streptomyces, Kocuria*, and *Pseudomonas* have also been reported as the dominant culturable bacterial genera in polluted air in Beijing during the heating season (Mao et al., 2019).

**Figure 3:**
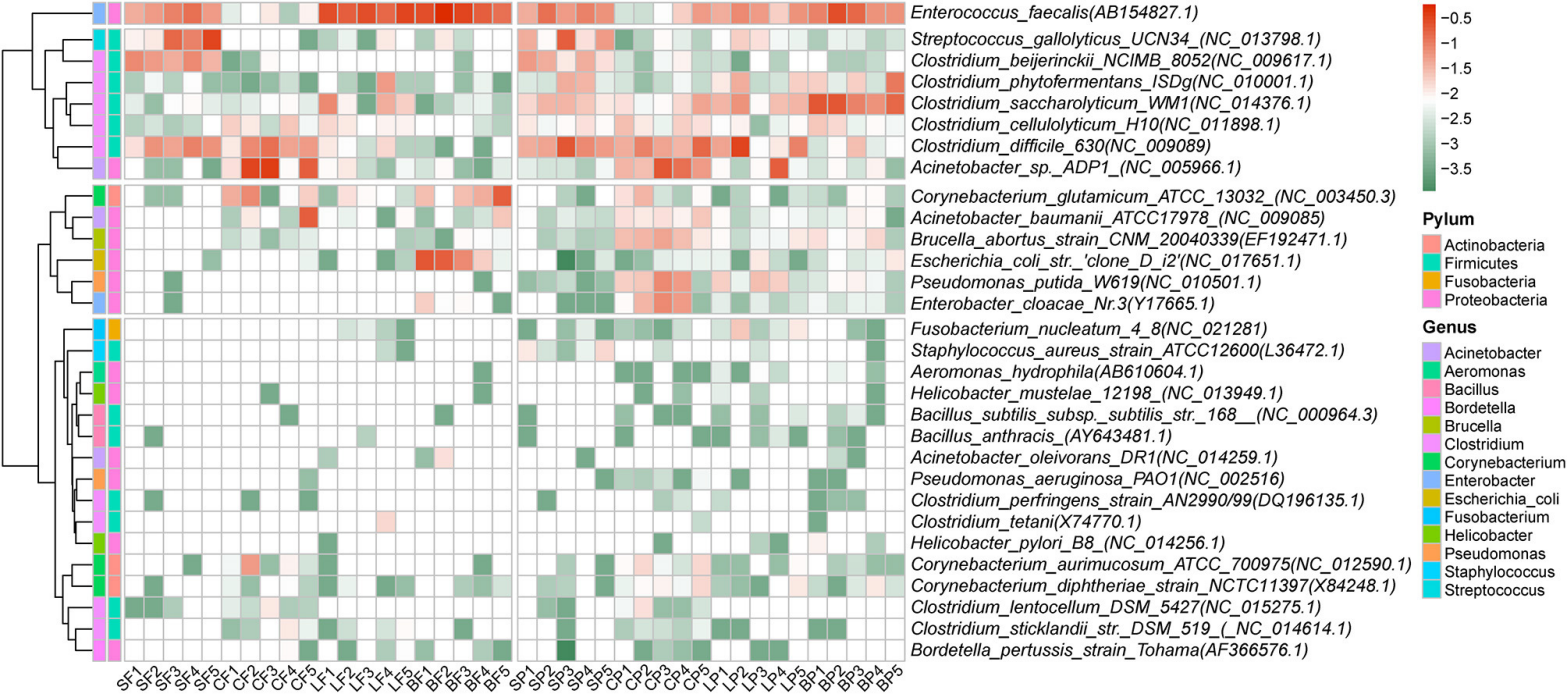
Relative abundance of dominant 30 human pathogenic bacteria in fecal and PM2.5 samples by animal species.

A total of 44 potential bacterial pathogenic species belonging to 22 genera were identified, and their total relative abundances in fecal and airborne bacteria were 39.7 ± 18.2% and 37.2 ± 13.4%, respectively. There were no significant differences in the distribution of human pathogenic bacteria in the feces and air of the animal farms. The top 30 species are shown in Figure 3. The prevalent pathogens were generally detected from *Enterococcus faecalis, Acinetobacter sp.*, and *Clostridium difficile* in both air and fecal samples. Other dominant pathogenic species in fecal and PM2.5 samples were *Streptococcus gallolyticus* (8.4 ± 3.3%) and *Clostridium saccharolyticum* (6.1 ± 4.2%), respectively. *Streptococcus gallolyticus* was more abundant in swine feces. It is a causative agent in ruminants and birds and may cause digestive disorders such as lactic acidosis (Sitthicharoenchai et al., 2022). The relative abundance of *Clostridium saccharolyticum* was high in all air samples in this study, and it was also detected in high abundance in poultry feces in a previous study (Luo et al., 2013). *Clostridium saccharolyticum* can cause intestinal and soft tissue infections in animals and humans (Luo et al., 2013).

The average relative abundance of 44 bacterial pathogenic species ranged from 0.002% to 16.3% in fecal samples and from 0.001 to 7.2% in air samples. Although no significant differences in the overall relative abundance among the four animals were detected in either feces or air samples (*p* = 0.08), some specific pathogenic species in layer and broiler farms (both in feces and air) were significantly higher than those in swine feces, such as *Enterococcus faecalis* (*p* < 0.05). *Acinetobacter sp*. was the most dominant pathogenic bacterium in cattle feces but was almost non-existent in swine feces. In this study, *Clostridium difficile* was abundant in swine and cattle feces, which confirmed that *C*. *difficile* is mainly distributed in the feces of large animals (Baverud et al., 2003). *Clostridium difficile* is a zoonotic bacterium (Rodriguez et al., 2016). Consumption of *C. difficile*-contaminated foods by vulnerable people with gastrointestinal disorders may lead to infection or asymptomatic transmission in the community (Rodriguez et al., 2016).

### 3.2. Aerosolization behavior of antibiotic resistance genes, antibiotic resistance bacteria, and human pathogenic bacteria

**Figure 4:**
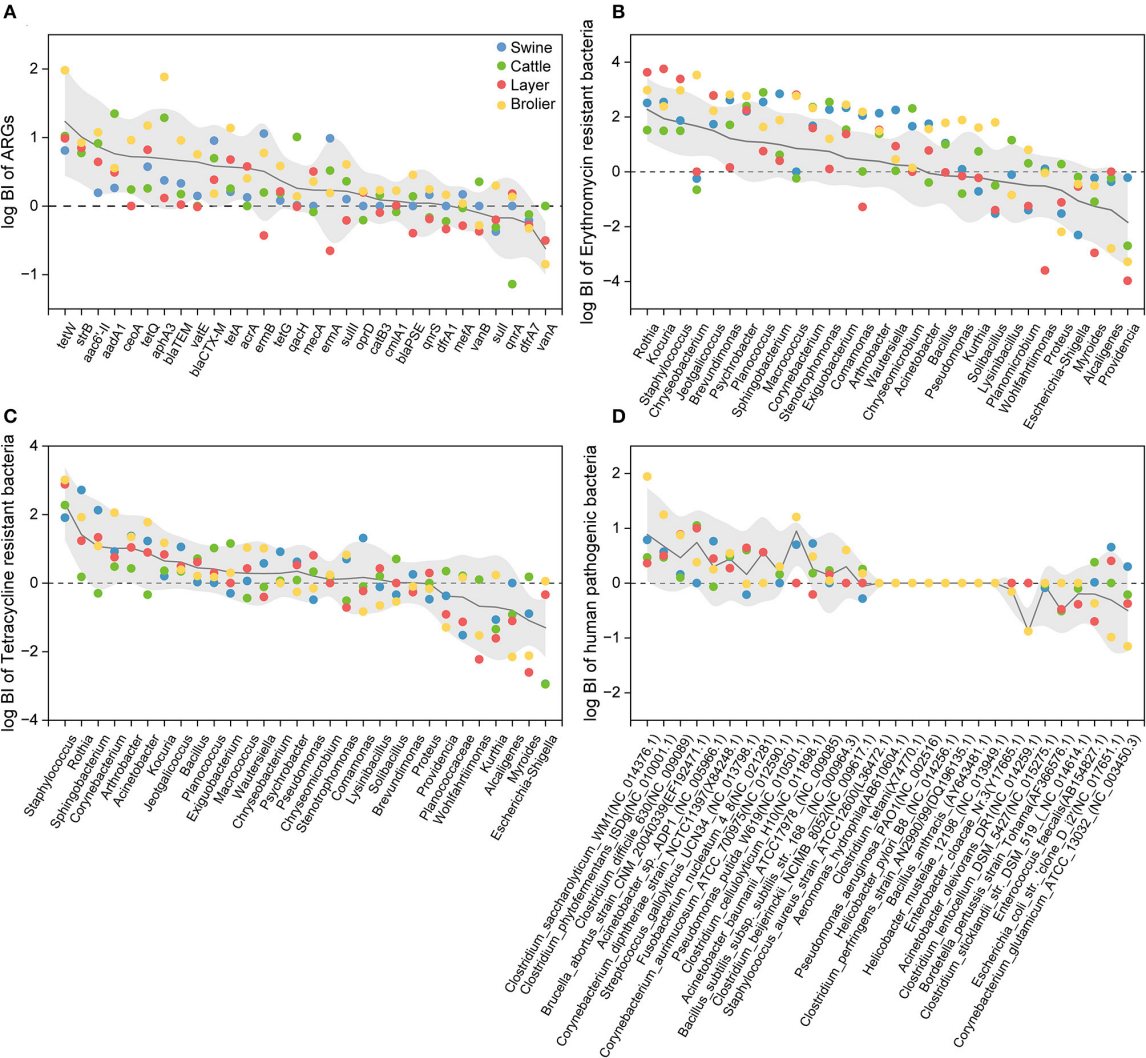
Bioaerosolization index (BI) of top 30 bacterial genera of ARGs **(A)**, erythromycin- **(B)** and tetracycline-resistant bacterial genera **(C)**, and top 30 human pathogenic bacteria **(D)**. The BI value was log-transformed.

In this study, the bioaerosolization index (BI) was used to indicate the aerosolization behavior of ARGs, ARBs, and HPBs in feces, and the distribution patterns of their corresponding BI values are shown in Figure 4. When the log BI values were >0, the targets were more likely to aerosolize from animal feces into the air.

The aerosolization behavior of ARGs in the feces is shown in Figure 4A. Overall, the BI values of the ARGs ranged from 0.04 to 460, and the highest (*tetW*) and lowest *(sulI*) BI values were detected in broiler and swine farms. Among the 30 ARGs investigated, 73.3% of the ARGs could be preferentially aerosolized (log BI > 0), in which the genes that encoded the aminoglycoside and tetracycline resistance were more likely to be aerosolized with the log BI values greater than 0. A similarly high relative abundance of ARGs in the air has been previously reported in wastewater treatment plants (Yang et al., 2018), indicating that the preferential aerosolization of certain ARGs may be a ubiquitous phenomenon. However, although *vanA* and *vanB*, which encode vancomycin (known as “a last-resort drug”), were detected in both feces and air, these two genes were mostly difficult to aerosolize (except *vanB* on the cattle farm). The current results indicate that when evaluating the potential contribution of animal feces to the surrounding airborne AMR, it is necessary to comprehensively consider both the concentration of target pollutants in potential sources and their corresponding aerosolization capacity.

The number of preferentially aerosolized ARGs varied by animal species in the following order: broilers (27), cattle (21), layer chickens (19), and swine (18). The highest BI value observed at broiler, layer, swine, and cattle farms were *tetW* (log BI = 1.97 ± 0.68), *tetW* (log BI = 1.23 ± 0.79), *ermB* (log BI = 1.06 ± 0.86), and *aadA1* (log BI = 1.35 ± 0.49), respectively. It is noteworthy that all the above genes are high-risk ARGs (Zhang et al., 2021), indicating their varying potential enhanced risk when transmitted through the air in different farms. We also found that the BI of the same ARG varied according to animal species. Macrolide-lincosamide-streptogramin B (MLSB) genes (*ermB, ermA*, and *mefA*) were difficult to aerosolize in layer farms (log BI < 0), whereas their log BI in other animal farms was > 0. The aerosolization behavior of FCA resistance genes also showed different trends in different animal farms, especially *qnrS* and *qnrA*. These two genes were classified as rank I high-risk ARGs and both showed passive aerosolization on cattle farms, whereas they were preferentially aerosolized on broiler farms. In contrast, the log BI values of *vanB* for vancomycin-resistance genes were greater than 0 only on cattle farms. This suggests that the aerosolization behavior of ARGs may be affected by the animal species, and the transmission risk caused by different types of animals is not completely consistent.

As shown in Figures 4B, C, the aerosolization of the top 30 erythromycin- and tetracycline-resistant bacterial genera were similar; more than half of the ARBs showed preferential aerosolization, with an average BI of 17 and 18. According to previous research on the aerosolization behavior of bacteria, 11 and 10 genera with BI values higher than 0 were detected in vegetable composting (He et al., 2019) and sludge composting processes (Lu et al., 2021), respectively. One possible explanation for the high BI value of ARBs is that some bacteria have been reported to become more resistant to harsh environments after acquiring antibiotic resistance (Yang et al., 2016). The increased survivability of ARBs in the air was reflected in the higher BI values in this study.

The preferred aerosolized erythromycin-resistant bacteria were *Rothia* (log BI = 2.28 ± 1.14). *Staphylococcus* shows preferential aerosolization behavior in both erythromycin- and tetracycline-resistant bacteria with log BI values of 1.94 ± 1.11 and 2.52 ± 1.02, respectively. This may explain the prevalence of *Staphylococcus* (Figure 2). Although *Myroides* and *Escherichia–Shigella* were the dominant bacterial genera in two types of fecal ARBs (Figure 2), it seemed that they were difficult to aerosolize in the animal farm with BI values lower than 0. The same bacterial genera with resistance to different antibiotics show distinguished aerosolized behaviors that the log BI value of erythromycin- and tetracycline-resistant *Acinetobacter* were−0.17 ± 0.09 and 0.88 ± 0.63, respectively. Although the characteristics of the bacterium itself are considered the main determinants of its aerosolization behavior (Lu et al., 2021), the results of this study indicated that other factors might also play a non-negligible role.

In terms of human pathogenic bacteria, 14 of the top 30 bacteria were prone to preferential aerosolization (log BI > 0). *Clostridium saccharolyticum WM1* was the most easily aerosolized pathogen in swine and broiler farms with log BI = 0.79 ± 0.50 and 1.94 ± 0.68, respectively. *Brucella abortus* strain *CNM 20040339* had the highest value at cattle and layer farms with log BI = 1.05 ± 0.49 and log BI = 1.01 ± 0.28. *Brucella abortus* mainly infects animals, such as swine and cattle, and some studies have found that it may damage the reproductive system and joints of humans and animals (Aliyev et al., 2022). *Brucella abortus* mainly infected cattle and sheep breeders, slaughterers, and processors, and has been shown to be transmitted by aerosols (Kahl-McDonagh et al., 2007).

The aerosolization behavior of the pathogens was also animal-specific. *Streptococcus gallolyticus* was preferentially aerosolized in cattle and layer farms, with log BI = 0.60 ± 0.31 and 0.64 ± 0.11. *Corynebacterium glutamicum ATCC 13032* gave priority to aerosols in swine farms but to passive aerosols in other animal farms. Among the 44 detected pathogenic bacterial species, some pathogens not only cause animal disease outbreaks and affect the production of meat or eggs but also pose potential risks to human health. For example, *Clostridium difficile* showed a high abundance in the air with preferential aerosolization behavior (mean log BI = 0.46 ± 0.32). As one of the most common pathogens in nosocomial infections, *Clostridium difficile* accounts for 15% of all pathogens associated with healthcare-related infections (Magill et al., 2018). The escape of *Clostridium difficile* from animal feces may pose a threat to the health of workers and residents around farms and even lead to community transmission. Preferentially aerosolized *Streptococcus gallolyticus* in cattle and layer farms also calls attention because it can cause meningitis, arthritis, and septicemia in pigs and meningitis and toxic shock syndrome in humans, which could lead to death in severe cases (Nguyen et al., 2021).

### 3.3. Potential factors affecting the aerosolization behavior of antibiotic resistance genes, antibiotic resistance bacteria, and human pathogenic bacteria

According to the distribution of BI involving ARGs, ARBs, and HPBs from the four types of animal farms (Figure 4), fluctuating values of the three types of targets were observed in different animal farms, even in the same target. In addition to the abovementioned animal types, it was assumed that other parameters might also affect the aerosolization process, including breeding-related parameters (age, animal number, breeding area, and stocking density) and environment-related parameters (temperature, relative humidity, and PM2.5). Details are described in our previous study (Xin et al., 2022).

**Figure 5:**
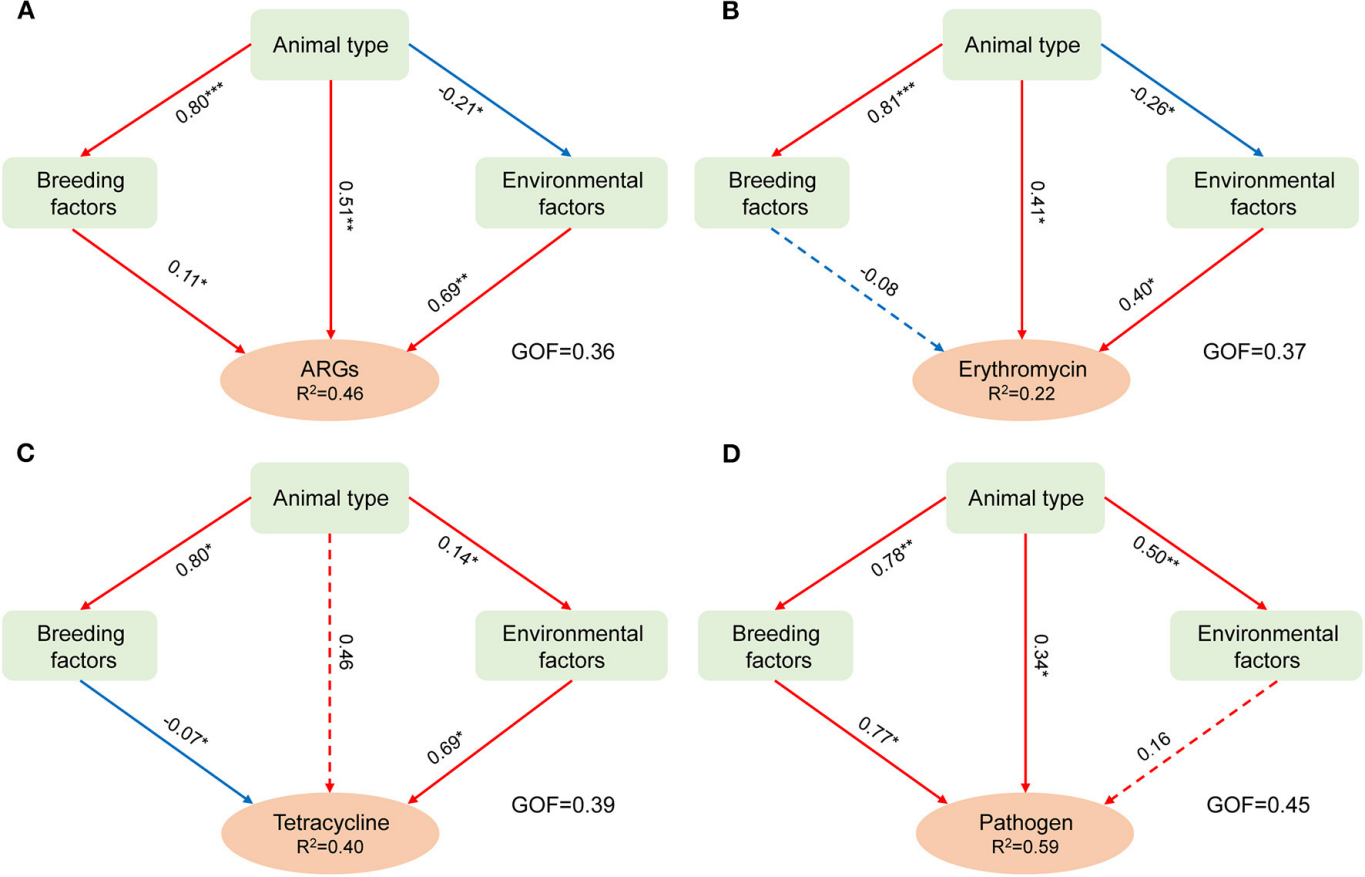
Main factors influencing the aerosolization behavior of antibiotic-resistance genes **(A)**, erythromycin-resistant bacterial genera **(B)**, tetracycline-resistant bacterial genera **(C)**, and human pathogenic bacteria **(D)** using partial least square path modeling (PLS-PM). The red and blue arrows represented the positive and negative effects, respectively. The dotted and solid lines indicated significance (*p ≤ 0.05, **p ≤ 0.01, ***p ≤ 0.001). GOF indicated the overall measure of model fit (Henseler and Sarstedt, 2013).

Partial least squares path modeling (PLS-PM) was used to differentiate the effects of breeding, environmental, and animal types on aerosolization behavior. As shown in Figure 5, the GOF of the PLS-PM model was greater than 0.36, indicating that the parameters examined in this study significantly affected the variation in BI values (Henseler and Sarstedt, 2013). Overall, the aerosolization behavior of the targets was significantly influenced by the animal type (except for the abundance of tetracycline-resistant bacteria), which confirmed the results discussed above. In addition, the positive correlations between environmental factors and the aerosolization behaviors of ARGs and ARBs were all significant. The influence on HPB was also positive, with a path coefficient of 0.16. The results indicated that increasing the temperature, relative humidity, and PM2.5 mass concentration in the air might promote the escape of microorganisms in feces. High values of environmental factors within the ranges used in this study increase the survival ability of organisms (Kathiriya et al., 2021). The two types of ARBs showed the same negative correlation with breeding-related parameters. Notably, all the factors investigated here can accelerate the aerosol process of ARGs and HPBs.

Previous studies have discussed the potential factors for bacteria or fungi escaping animal manure composting (Wang R. et al., 2022) and vegetable waste composting (He et al., 2019), mainly focusing on the physicochemical properties of the composting pile, such as the concentration of heavy metals and other influencing factors (such as C/N, DOC, and pH) on aerosolization behavior. The results of this study improved the role of influencing factors on the aerosol behavior of four types of targets based on a comprehensive discussion of the possible effects of environmental and breeding-related parameters, which provides an important reference basis for the targeted control of the escape of AMR elements and HPBs.

**Figure 6:**
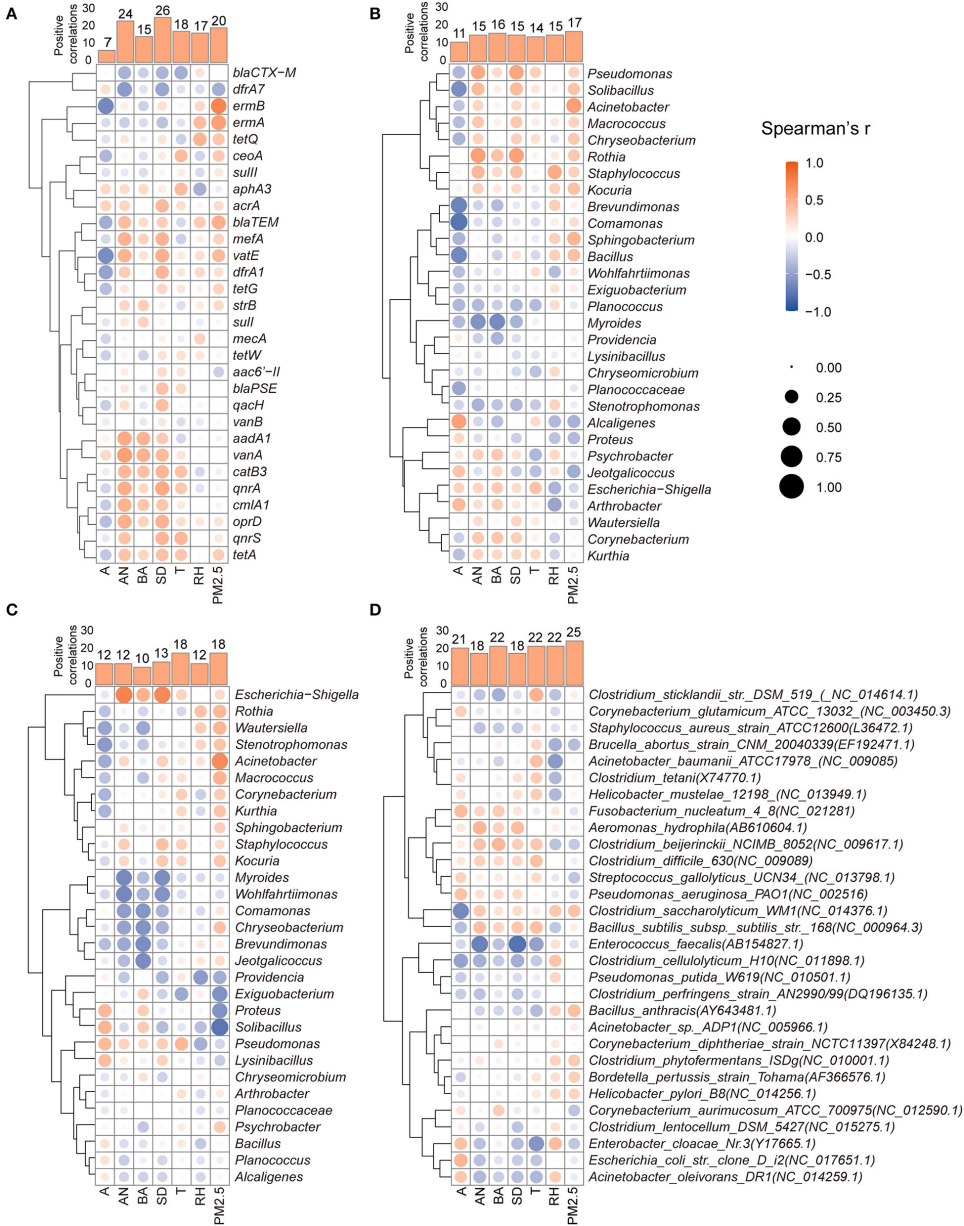
Factors associated with the aerosolization behavior of specific ARGs **(A)**, erythromycin-resistant bacterial genera **(B)**, tetracycline-resistant bacterial genera **(C)**, and human pathogenic bacteria **(D)**. (A, Age; AN, animal number; BA, breeding area; SD, stocking density; T, temperature; RH, relative humidity; PM2.5, PM2.5 mass concentration).

To clarify the specific factors affecting the aerosolization behavior of dominant targets, the relationships between each parameter and the BI value of ARGs, ARB, and HPB were further analyzed using a correlation heatmap and Spearman's correlation coefficient as shown in Figure 6. According to the relationship between the seven factors and the BI of 30 ARGs in Figure 6A, animal number (24) and stocking density (26) were positively correlated with the aerosolization of the majority of genes, which were usually the subtypes with high concentrations in animal feces (Figure 1). These results were observed mainly in poultry farms. Chicken farms with large depositories or high breeding densities usually have a high cleaning frequency, which may promote the escape of ARGs (Liu et al., 2018). Animal age mostly negatively affected the BI of ARGs (Figure 6A) and two types of ARBs (Figures 6B, C), which may be related to the decreasing of fecal bacterial AMR as animal breeding time (Berge et al., 2005; Gaire et al., 2022).

As shown in Figures 6B, C, the potential factors had a relatively neutral impact on the BI value, and no obvious promoting or inhibiting trends were found in this study. The number of positive correlations between the BI and temperature (18) was comparable to that between the BI and humidity (17). However, the effects of temperature and relative humidity on the BI values of the same ARGs showed opposite trends. This may be due to the interactive effects of temperature and relative humidity on bioaerosol activity (Dungan, 2010), which may inactivate microbial proteins under their synergistic influence. Although the effects of temperature and relative humidity on bacterial aerosolization require further investigation, the results of the present study suggest that farms can regulate air temperature and relative humidity to control the aerosolization behavior of airborne bacteria. In addition, some breeding-related parameters, such as the animal number and stocking density, had similar effects on certain types of ARBs. For example, erythromycin- and tetracycline-resistant *Escherichia–Shigella, Staphylococcus, Kurthia*, and *Pseudomonas* had positive correlations with the animal number, breeding area, and stocking density.

For HPBs, the results of the correlation heatmap (Figure 6D) showed that most parameters were positively correlated with the aerosolization behavior of HPBs; in particular, the PM2.5 mass concentration was positively correlated with the BI of 25 HPBs. Overall, PM2.5 mainly promotes the biological aerosolization process. Previous studies have shown that the abundance and diversity of airborne ARGs in the environment increase correspondingly with PM2.5 mass concentrations (Sun et al., 2020). The air environment on animal farms is filled with various pollutants, including both inert and active substances, which may provide protection and nutrition to airborne microorganisms, resulting in increased survivability in the air (Sun et al., 2020). Previous studies have shown that the proportion of pathogenic bacteria (*Streptococcus pneumoniae*) increases with increasing PM levels (Cao et al., 2014), and the increased survival of microorganisms in the air is reflected in the increased BI value. For preferentially aerosolized HPBs (BI > 0), the aerosolization behaviors of *Staphylococcus aureus* and *Escherichia coli* were mainly positively correlated with RH and PM2.5, and *Pseudomonas aeruginosa* and *Acinetobacter baumannii* were mainly positively correlated with breeding factors.

## 4. Conclusion

Based on the analysis of the aerosolization behavior and influencing factors of ARGs, ARBs, and HPBs in animal feces from four types of animal farms, the results of this study showed that most of the targets in animal feces were aerosolized, which included human pathogenic bacteria and high-risk ARGs. The average bioaerosolization index of ARGs, ARBs, and HPBs in broiler farms was generally the highest, whereas that in cattle farms was the lowest. Breeding-related parameters had a much more significant effect on aerosolization. The results will help comprehensively assess the potential health risks of fecal microorganisms transported by air paths and their contribution to the surrounding environment. At the same time, it provides technical parameters for the targeted control of the escape of pathogenic bacteria and high-risk ARGs.

## Data availability statement

The datasets presented in this study can be found in online repositories. The names of the repository/repositories and accession number(s) can be found in the article/supplementary material.

## Author contributions

HX: writing—original draft preparation, investigation, and formal analysis. TQ and YG: investigation and data curation. HG: investigation and software. LZ: writing—review and editing and conceptualization. MG: writing—review and editing, project administration, and funding acquisition. All the authors contributed to the manuscript and approved the submitted version.
